# Downregulation of MCT4 for lactate exchange promotes the cytotoxicity of NK cells in breast carcinoma

**DOI:** 10.1002/cam4.1713

**Published:** 2018-07-26

**Authors:** Yaping Long, Zihe Gao, Xiao Hu, Feng Xiang, Zhaozhen Wu, Jiahui Zhang, Xiao Han, Liyong Yin, Junfang Qin, Lan Lan, Fuzai Yin, Yue Wang

**Affiliations:** ^1^ School of Medicine Nankai University Tianjin China; ^2^ First Hospital of Qinhuangdao Qinhuangdao Hebei China; ^3^ Tianjin Cancer Hospital Tianjin Medical University Tianjin China; ^4^ State Key Laboratory of Medicinal Chemical Biology NanKai University Tianjin China

**Keywords:** lactate, MCT4, NK cytotoxicity, NKG2D, NKG2DLs (H60)

## Abstract

Monocarboxylate transporter‐4 (MCT4), a monocarboxylic acid transporter, demonstrates significantly increased expression in the majority of malignancies. We performed an experiment using BALB/C mice, and our results showed that ShMCT4 transfection or the pharmaceutic inhibition of MCT4 with 7acc1 strengthens the activity of NK cells. The results of a calcein assay revealed that the cytotoxicity of NK cells was strengthened via inhibition of MCT4. In addition, ELISA testing showed that the content of perforin and CD107a was increased, and PCR amplification and immunoblotting revealed that the expression of NKG2D and H60 was upregulated after the inhibition of MCT4. Further, we observed an elevated pH value, decreased extracellular lactate flow, and attenuated tumor growth. Therefore, we concluded that the inhibition of MCT4 enhanced the cytotoxicity of NK cells by blocking lactate flux and reversing the acidified tumor microenvironment. In addition to these findings, we also discovered that MCT4 depletion may have a pronounced impact on autophagy, which was surmised by observing that the inhibition of autophagy (3MA) pulled the enhanced cytotoxicity of NK cells downwards. Together, these data suggest that the key effect of MCT4 depletion on NK cells probably utilizes inductive autophagy as a compensatory metabolic mechanism to minimize the acidic extracellular microenvironment associated with lactate export in tumors.

## 1 INTRODUCTION

Glycolysis produces abundant lactic acid as the main method of harvesting energy, which enables malignant tumor cells to survive in anoxic microenvironments.^1^, ^2^ Because decreasing the extracellular pH (pHe) changes the immune phenotype and disturbs the immune function of tumor infiltrating lymphocytes, the enhanced lactic acid is more conducive to tumor immune escape and invasion.^3^, ^4^, ^5^, ^6^, ^7^ The pH values of tumor tissues are usually between 7.2 and 6.8 or even as low as 5.6.^8^, ^9^ The lower pH may mask the efficacy of chemotherapy by altering drug distribution and metabolism. This lower pH may also skew T and NK cells toward the immunosuppressive phenotype and function.^10^, ^11^, ^12^, ^13^ The cytotoxicity of NK cells is detected to be impaired by 50% when providing an acidic microenvironment in which the pH values vary from 7.2 to 7.0. NK cell cytotoxicity may even drop to 0% if the pH value decreases to 6.5.^14^ Therefore, we hypothesized that reversing an acidified microenvironment might be an effective method to rescue the cytotoxicity of NK cells. Monocarboxylic acid transporter 4 (SLC16A3/MCT4) is one of the membrane protein stubs in cells that is structurally able to output lactic and pyruvic acids and affect the acidity of the tumor microenvironments.^15^, ^16^, ^17^ High expression of MCT4 has been reported to be a biomarker in various types of tumors,^18^, ^19^, ^20^, ^21^, ^22^, ^23^, ^24^ which makes it an appealing therapeutic target in cancers. The blockade of MCT4 expression has been shown to represent a novel treatment target in glioblastoma, large B‐cell lymphoma, and multiple myeloma.^25^, ^26^, ^27^ For these reasons, and because MCT4 has the capacity to alter the microenvironment in tumors, we felt we had a compelling reason to investigate the effect of MCT4 on NK cells.

Our study offered some important insight into the distribution of activated NK cells in human breast cancer tissues and provided an opportunity to further exploit the function of MCT4 on the lactic acid concentration and the cytotoxicity of NK cells in vitro and in vivo. This study also provided further insight regarding the relationship between tumor metabolism‐related molecules and immune‐effective cells. In this study, we demonstrated how MCT4 effectively influences the concentration of lactate and the cytotoxicity of NK cells.

## 2 MATERIALS AND METHODS

### 2.1 Cell culture and reagents

BALB/c female mice (16‐22 g) were obtained from the Academy of Military Medical Sciences in China. 4T1 cells were purchased from the Cancer Hospital Chinese Academy of Medical Sciences. TRIzol and DNA markers were purchased from TIANGEN BIOTECH (Beijing) Co., LTD. RPMI 1640 culture medium was purchased from Gibco Thermo Fisher Scientific. The mice spleen NK cell isolation optimizing system was purchased from Tianjin Haoyanghuake Biologic Product Co., LTD. 7acc1 (7‐aminocarboxycoumarin derivatives) was obtained from MCE, Shanghai, China. 3MA was purchased from Sigma, St. Louis, MO, USA. Calcein AM was acquired from Dojindo Molecular Technologies, Inc, Shanghai, China. L(+)‐lactic acid was purchased from Solarbio (L8630), Beijing, China.

### 2.2 Animal experiments

Animal experiments were performed according to the Guidelines on Laboratory Animals of Nankai University and were approved by the Institute Research Ethics Committee at Nankai University. Animals were housed in 330 × 215 × 200 mm cages (CHICO.J.X, China, Type IVC), given access to mice maintenance food (Xietongyiyao, Jiangsu, China, SKBT001) and kept in an SPF‐level lab. During housing, no adverse events were observed.

The tumor (mouse breast invasive ductal carcinoma)‐bearing BALB/c mice (n = 12) were established by inoculating subcutaneously with 1 × 10^6^ 4T1 breast cancer cells for 7 days; these mice were syngeneic to BALB/c mice and were randomly divided into four groups (three/group), which were treated by a blind intraperitoneal (ip) injection of a multiple proportion dilution of 7acc1 (0.3, 0.03, 0.003, and 0 mg/kg as control group) each day for 7 days.

Additionally, 1 × 10^6^ 4T1 cells were coincubated with different concentrations of 7acc1 (0.1, 0.01, 0.001, and 0 mmol/L as control) for 24 hour, inoculated subcutaneously (s.c.) to the axilla of mice (n = 12, four groups, 3/group) and normally fed for 7 days.

Further, 1 × 10^6^ 4T1 cells with stable expression of MCT4 short hairpin RNA (ShRNA; relatively weak, medium, strong, and null vector transfection as the control) were inoculated s.c. to the axilla of mice (n = 12, four groups, 3/group) normally fed for 21 days when they became moribund to establish stable MCT4‐knockdown 4T1 by the vector‐based RNAi system. RNAi‐mediated MCT4 knockdown was accomplished by ShRNAs produced by the DNA‐based ShRNA‐expressing retroviral vector (pSuper‐Retro). After transfection, 4T1, which could stably express MCT4 ShRNA, was selected by ampicillin treatment (RiboBio Co., LTD, Guangzhou China).

### 2.3 Eyeball blood extraction

Blood taken by eyeball removal was kept in a 37°C water bath for 1 hour and centrifuged at 1789 g for 10 minutes. The serum was transferred to centrifuge tubes for ELISA analysis.

### 2.4 RNA isolation, reverse transcription, and real‐time quantitative PCR (RT‐ and qPCR)

Aliquots of 1 μg total RNA was reverse transcribed using TransScript First‐Strand cDNA Synthesis Supermix (TranGen Biotech, Beijing, China). qPCR was carried out with TransScript Top Green qPCR Supermix (TransGen, Beijing, China) using the Bio‐Rad CFX Manager System (Bio‐Rad, USA). The sequences of specific primers (Sangong Biotech, Shanghai, China) for PCR were as follows: *MCT4* (For, 5′‐GCCACCTCAACGCCTGCTA‐3′; Rev, 5′‐TGTCGGGTACACCCATATCCTTA‐3′), *NKG2D* (For, 5′‐ACGTTTCAGCCAGTATTGTGC‐3′; Rev, 5′‐GGAAGCTTGGCTCTGGTTC‐3′), *H60* (For, 5′‐GCCTCAACAAATCGTCAT‐3′; Rev, 5′‐ATACACCAAGCGAATACC‐3′), *LC3* (For, 5′‐CATGAGCGAGTTGGTCAAGA‐3′; Rev, 5′‐TTGACTCAGAAGCCGAAGGT‐3′), *Beclin‐1* (For, 5′‐GTTGCCGTTATACTGTTCTG‐3′; Rev, 5′‐CCTCCAGTGTCTTCAATC‐3′), and β*‐actin* (For, 5′‐CGTTGACATCCGTAAAGACC‐3′; Rev, 5′‐AACAGTCCGCCTAGAAGCAC‐3′). RT‐PCR was carried out using MG96G PCR instrumentation (LongGene, Hangzhou, China). The final results were analyzed by ImageJ2x.

### 2.5 Immunohistochemistry, immunofluorescence, and immunoblotting

Samples of hyperplasia in mammary glands and breast cancers were obtained from BinHai Hospital Peking University and coded anonymously in accordance with local ethical guidelines. Mouse breast cancer sections were acquired from the tumor‐bearing mice and were made into biopsies by histotome (Eastman Kodak Company, German). Paraffin‐embedded and formalin‐fixed samples were cut into 5 μm sections. The sections were exposed to 3% H_2_O_2_ and blocked with 5% sheep serum for 15 minutes, then incubated with anti‐CD56 (human, ZSGB‐BIO), anti‐NKG2D (human, BioSS), anti‐MCT4 (mouse, Millipore), anti‐NKG2D (mouse, Biolegend), anti‐H60 (mouse, Biolegend), anti‐LC3 (mouse, MBL), and anti‐Beclin‐1 (mouse, Santa Cruz) antibodies at 4°C overnight, and after that, incubated with a secondary antibody. Finally, the visualization of immune complexes was performed by diaminocarbazole (DAB) and quantified by Image‐Pro Plus 6.0. The measurements were expressed in densities (IOD/Area).

For the immunofluorescence staining analysis, the sections were stained with monoclonal mouse anti‐mouse MCT4 (Millipore) (1:200), rabbit anti‐mouse NKG2D (Biolegend) (1:200), and rabbit anti‐mouse H60 (Biolegend) (1:200), followed by FITC‐conjugated goat anti‐mouse IgG (H + L), TRITC‐conjugated goat anti‐mouse IgG, and PE‐conjugated goat anti‐rabbit IgG (H + L) (1:100, ZSGB‐BIO, Beijing, China). Nuclei were stained with DAPI. Images were viewed and assessed using a confocal microscope (Olympus, FV1000).

For the Western blot analysis, whole proteins were loaded into the lanes of SDS‐polyacrylamide gels and separated by electrophoresis. Then, the proteins were transferred to PVDF membranes and probed with mouse anti‐mouse MCT4 (Millipore) (1:200), rabbit anti‐mouse NKG2D (Biolegend) (1:200), rabbit anti‐mouse H60 (Biolegend) (1:200), and β‐actin (1:3000, Santa Cruz Biotechnology). β‐actin was detected as a loading control. The consequence was analyzed by ImageJ2x.

### 2.6 ELISA

Mice were sacrificed after 4T1 inoculation treatment, and the serum was isolated from blood samples by eyeball extirpating and then was used for concentration detection of LAMP‐1 (CD107a) (ElabScience) and perforin 1 (PRF1) (ElabScience) following the kit's protocol. All the assays were performed in triplicate.

### 2.7 Cytotoxicity assay

The 4T1 cells were treated with 7acc1 or 3MA and incubated with calcein AM. Then, the cells were incubated with freshly isolated NK cells extracted using an NK Cells Isolating Kit (TBD Science, Tianjin, China) for 4 hour at various effector/target ratios (50:1 and 100:1). Other 4T1 cells incubated with calcein AM were treated with lactate (Solarbio) and incubated with freshly isolated NK cells as above. The fluorescence of each supernatant was measured at 490 nm excitation and 515 nm emissions using the Multiscan Spectrum. The following calculation was used in the analysis:

$$\begin{array}{cc} & \%\;\text{specific lysis}\\ & =(\text{experimental OD}-\text{spontaneous OD})\\ & \;/(\text{maximum OD}-\text{spontaneous OD})\times 100\%. \end{array}$$

### 2.8 Extracellular pH measure

The pH value of the culture medium supernatant was measured by PP‐15‐P11 Sartorius pH‐Meters (Germany). The pH detection range was −2.000 to 20.000. The pH default resolution and confidence were ±0.001. All samples were assayed three times.

### 2.9 Lactate assay

The 4T1 cells were seeded in medium and allowed to recover overnight. Cultures were then treated with DMSO (0.001, 0.01, and 0.1 mmol/L) and 7acc1 and placed for 4, 8, and 12 hours. The cell supernatant was taken to measure with the Mouse Lactate ELISA Kit (Nanjing Jin Yibai Biological Technology Co., Ltd., Nanjing, China). The ODs at a wavelength of 450 nm were analyzed by Curve Expert 1.4.

### 2.10 Statistics

All results were presented as the mean ± SEM. Statistical analyses were performed with the Wilcoxon rank test and Student's *t* test, and differences with *P *<* *0.05 were considered significant. The cytotoxicity assay OD and pH values were analyzed using a LOESS regression analysis, from which the regression curve and regression formula (*y*~*x*) were obtained. The shadow area on either side of the curve expresses the 95% confidence interval.

## 3 RESULTS

### 3.1 Lack of an adequate NKG2D expression pattern was manifested in the human breast cancer tissues

**Figure 1:**
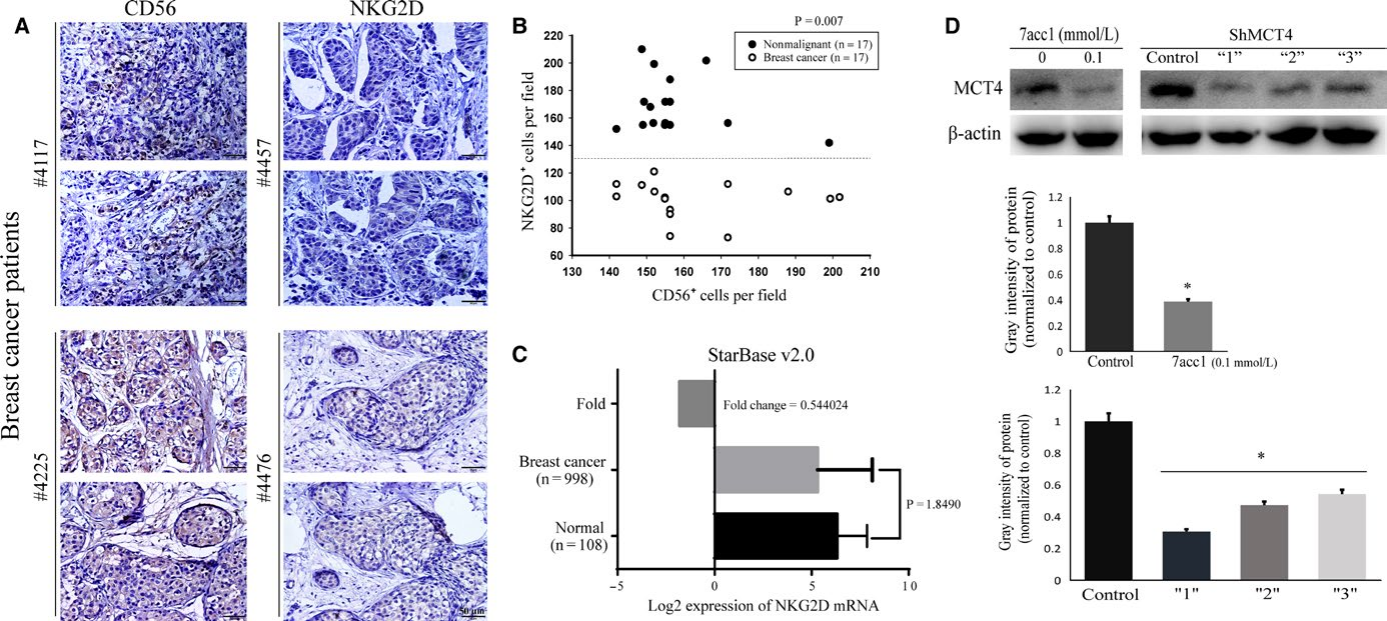
NKG2D deficiency was identified in human breast cancer tissues, and MCT4 expression was detected after 7acc1 (a MCT4 inhibitor) treatment and ShMCT4. A, Representative images of CD56 and NKG2D expression detected by immunohistochemistry in four randomly selected breast cancer patients’ tissues. B, Statistical analyses of the CD56^+^ and NKG2D^+^ cell densities in the breast cancer tissues and the nonmalignant hyperplasia tissues from the patients. C, NKG2D mRNA levels in 1106 samples from breast cancer and normal breast tissues were analyzed using the starBase Pan‐Cancer Analysis Platform. D, The protein expression of MCT4 in the murine breast cancer cell line 4T1 treated with 7acc1 (0.1 mmol/L) or transfected with different ShMCT4 vectors (weak “1,” medium “2,” and strong “3”). *P <* *0.05

Using the IHC assay in human breast cancer tissues, we found a pronounced decline in the quantity of activated NK cells infiltrating the tumor. To estimate the distribution of the activated NK cells, human breast cancer tissue biopsies were analyzed. As NKG2D is the major cytotoxicity receptor,^28^ we examined the expression of CD56 and NKG2D in the routine paraffin sections of human breast tissues. The distributions of CD56^+^ and NKG2D^+^ NK cells were established using DAB staining. This experiment was divided into two groups (the mammary gland hyperplasia patients’ specimens, n = 17; the breast cancer patients’ specimens, n = 17, collected in the Pathology Department of the hospital). We found that in the tissues shown in Figure 1A, which were obtained from four randomly selected breast cancer patients, there was not a lack of CD56‐positive cells but a lack of NKG2D‐positive cells instead (Figure 1A). Compared with the noncancerous tissue samples, the number of NKG2D^+^ (a marker for the cytotoxicity of NK cells) cells distributed in the breast cancer tissues had decreased significantly (*P <* *0.01 vs. the nonmalignant tissues, Figure 1B). The distribution of CD56^+^ (marker for the number of NK cells) cells in the cancer tissues, however, displayed no significant difference (*P *>* *0.05, Figure 1B) versus the nonmalignant tissues. The results showed that in the malignant tissues, although there was not a shortage in the number of NK cells, they had a lack of cytotoxicity when they were exposed to the carcinoma nests. The mRNA expression pattern of NKG2D in the 1023 breast cancer cases drawn from multiple types of 6630 human cancer patient studies cataloged in the *starBase v2.0* validated a decreased expression of NKG2D mRNA (Figure 1C). The results further confirmed that NKG2D was defectively expressed in malignant breast tissues.

### 3.2 Inhibition of MCT4 elevated the cytotoxicity of NK cells in vivo

In this study, we attempted to determine whether blocking MCT4 could alter the tumor microenvironment to improve NK cell cytotoxicity. To implement the research, we first identified a specific MCT4 inhibitor, 7acc1 (Figures 1D and S1). To determine the potential effects of MCT4 on the tumorigenic growth of 4T1 breast cancer cells, we employed stable short hairpin RNA (ShRNA)‐mediated knockdown. Retroviral infection of ShRNA limits the expression of MCT4 in 4T1 cancer cell lines. Therefore, stable ShMCT4 was utilized to effectively suppress the expression of MCT4 (Figure 1D).

**Figure 2:**
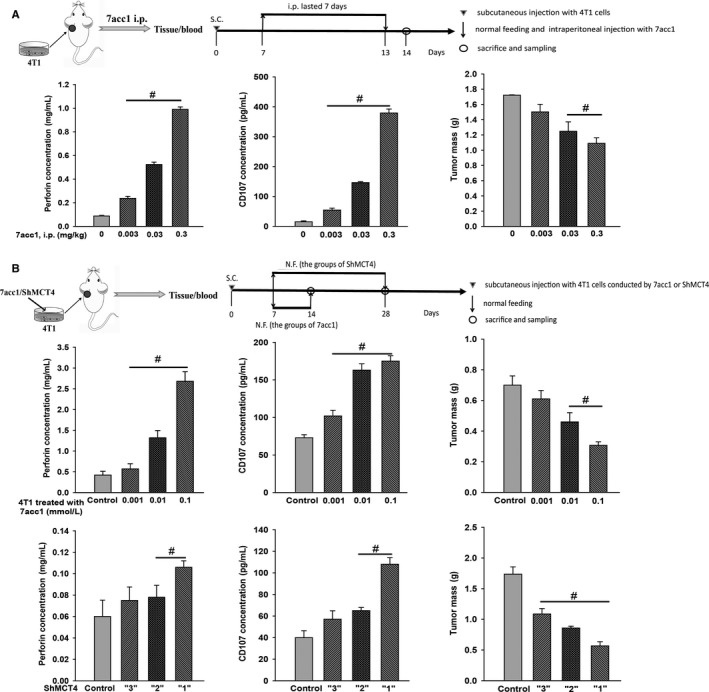
Both 7acc1 and ShMCT4 inhibited breast cancer growth and downregulated the secretion of cytotoxic related molecules (perforin and CD107) of NK cells in the blood of Balb/c mouse breast tumors. A, Perforin, CD107, and tumor mass were measured from the tumor‐bearing mice following intraperitoneal (ip) injection once per day for 7 d with different doses of 7acc1 (0, 0.003, 0.03, and 0.3 mg/kg). B, 4T1 cells pretreated with different doses of 7acc1 or ShMCT4 vectors (weak “1,” medium “2,” and strong “3”), then were inoculated in Balb/c mice. Tumor masses and the blood from these animals were harvested at different times as shown in the schematic diagrams. ^#^ *P *<* *0.05

Different doses of 7acc1 (0.3, 3 × 10^−2^, 3 × 10^−3^, and 0 mg/kg) (Figures 2A and S2) were applied to the tumor‐bearing mice by intraperitoneal (ip) injection for 7 days. The mice were sacrificed after tumorigenesis; eyeball blood was extracted, and the tumors were weighed individually. The inhibition effect of 7acc1 on tumor growth in vivo was then evaluated by weighing the tumor. Within the appropriate concentration range, with the elevated dose of 7acc1 applied, the mass of the solid tumor was significantly reduced.

Perforin and CD107a (lysosomal‐associated membrane protein‐1, LAMP‐1) were employed as two markers of NK cell functional activity.^29^, ^30^ To confirm the correlation between the impact of MCT4 inhibitor on tumor growth and the functional activity of NK cells, an evaluation of CD107a and perforin concentration was conducted by ELISA. As the efficiency of MCT4 inhibition increased, we observed a significant upregulation of perforin and CD107a, along with a decrease in tumor weight (*P <* *0.05 vs. the control group without the drug, Figure 2A). Similar characteristics of differentiation were obtained from the 7acc1 delivery methods for 14 days (data not shown).

**Figure 3:**
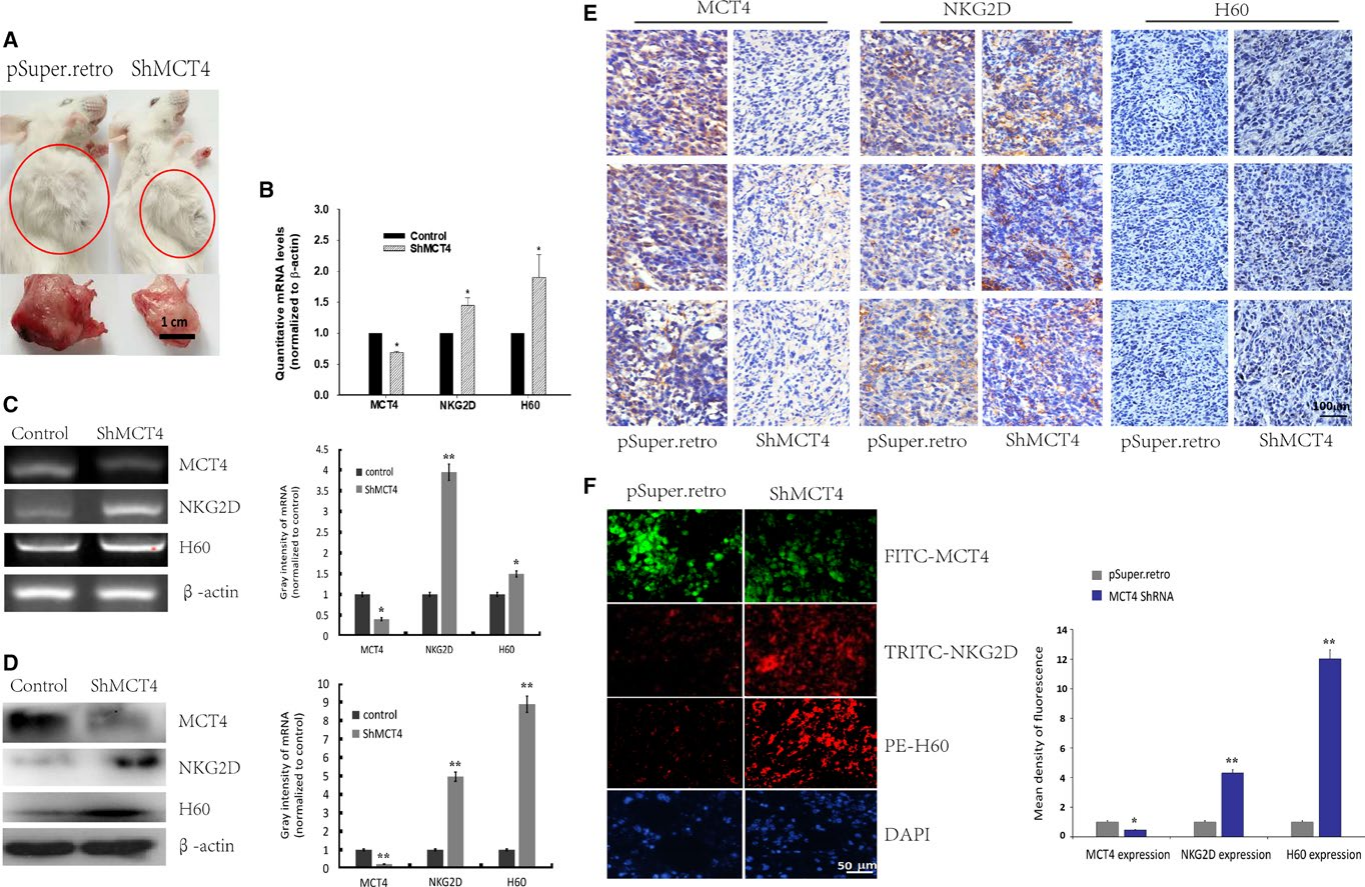
Deficiency of MCT4 promoted the expression of NKG2D and H60 in the tumor tissues. Tumor masses from mice inoculated with 4T1‐ShMCT4 “1” cells and 4T1‐Psuper.retro cells were harvested, and MCT4/NKG2D/H60 expression levels were determined. A, Representative images of tumor mass; B‐D, Quantitative, semiquantitative mRNA, or protein expression of MCT4/NKG2D/H60 by real‐time PCR, RT‐PCR, or Western blot analysis, respectively. E and F, MCT4/NKG2D/H60‐positive cells were stained by immunohistochemistry and immunofluorescence. Decreased MCT4 expression was accompanied by enhanced expression of NKG2D and H60, which are hallmarks of NK cytotoxicity, through modulating receptor‐ligand interaction of NK cells. * *P *<* *0.05

We also treated 4T1 cells with different concentrations of 7acc1 (0.1, 0.01, 0.001, and 0 mmol/L) overnight (Figure 2B and S2), followed by subcutaneous (s.c.) injection into the mice. The same indicators were tested, which reproduced the finding that CD 107a and perforin concentrations increased with an increase in the concentration (*P <* *0.05 vs. the placebo control group, Figure 2B). Further, with ShMCT4 vectors, the 4T1 cells expressing MCT4 were obtained by transfection, and in accordance with the inference efficiency, were divided into groups “1,” “2,” and “3.” When the transfected 4T1 cells were injected subcutaneously (s.c.) into the mice, we noticed that as the silence efficiency declined, the tumors diminished in size in the ShMCT4 groups; the concentration of the CD107a and the perforin decreased and the weight of the tumor increased at the same time (*P <* *0.05 vs. the control group of with null vector transfection, Figures 2B and 3A).

### 3.3 Downregulation of MCT4 promoted NKG2D‐NKG2DLs

H60, a main ligand of the NKG2D receptor, was selected to determine if downregulation of MCT4 promoted NKG2D‐NKG2DLs. While analyzing the correlation among the NKG2D receptors, the mice tumor homogeneity at the mRNA (using RT‐PCR and real‐time PCR) and protein levels (Western blot) were also investigated. When the MCT4 expression declined, the receptor NKG2D and the ligand H60 were noted to be significantly upgraded at both the mRNA and protein levels (*P <* *0.05, **P <* *0.01 vs. the control group with null vector transfection, Figure 3B, C, D). Notably, when mice tumor tissue slices were obtained for morphological detection, they showed markedly increased H60 and NKG2D expression. In addition, the H60‐ and NKG2D‐positive cell numbers in the ShMCT4 groups were prominently elevated, which was consistent with mRNA and protein expression levels (Figure 3E,F, *P <* *0.05 vs. the control group with null vector transfection).

### 3.4 Inhibition of MCT4 enhanced the cytotoxicity of NK cells

**Figure 4:**
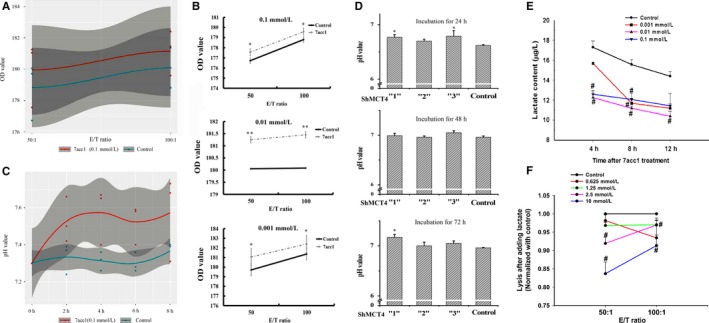
Inhibition of MCT4 enhanced the cytotoxicity of the NK cells via raising pH values and decreasing the lactic acid flux content of coculture cell supernatants. A, OD value was measured using the calcein AM release assay and calculated by Wilcoxon's test for the control and 7acc1 (0.1 mmol/L)‐treated groups. B, OD value was calculated at the different concentrations of 7acc1 (0.1, 0.01, or 0.001 mmol/L) treatment. The 7acc1 treatment groups (the dotted lines) enhanced 4T1 lysed by purified primary NK cells. A calcein AM cytotoxicity assay was performed using purified NK cells as the effector, effector: target ratio = 50:1 and 100:1. C, The pH values were measured using pH meters every 2 h and analyzed by Wilcoxon's test for the control and 7acc1 (0.1 mmol/L)‐treated groups. D, The pH values were described at different incubation time points (24, 36, and 72 h) and different interference ShMCT4 vectors (weak “1,” medium “2,” and strong “3”) in 4T1‐ShMCT4 and 4T1‐Shcontrol groups. After this shift, the culture supernatant pH value increased by 0.1 to 0.4 pH units; these results were consistent and repeatable among biological replicates. *P <* *0.05. E, Medium (0.7 ml) was obtained 24 h from the indicated cell after incubation with control or 7acc1. The concentration of lactate in the supernatant of 4T1 cells was determined using an ELISA kit. F, Freshly isolated primary NK cells were plated at the indicated ratios with calcein AM‐labeled 4T1 cells; then, the cells were stimulated by different concentrations of lactate. The cytotoxicity of the NK cells was determined. ^#^ *P *<* *0.05

To verify whether the cytotoxicity of NK cells was enhanced, primary NK cells were recruited and planted at the 50:1 and 100:1 ratios of effective cells to target cells (E/T ratio) along with 4T1 cells into 96‐well plates to undergo culture. We processed the target 4T1 cells with the 7acc1 at different concentrations (0.1, 0.01 and 0.001 mmol/L). By calcein AM assay, the OD values were calculated by monitoring calcein emissions. The 7acc1 treatment resulted in more calcein released into the supernatant, indicating a significant boost in the cytotoxicity of NK cells (the dotted lines shown). Impressively, the 7acc1 (0.1 mmol/L)‐treated group significantly increased by definition of the Loess regression curve (Figure 4A), and variance analysis of each incubation concentration of 7acc1 was also displayed (*P <* *0.05, **P <* *0.01 vs. the control group without the drug, Figure 4B). After blocking MCT4, the NKG2D‐NKG2DLS was significantly upregulated. Therefore, it is possible that this intervention may play a pivotal role in enhancing the overall cytotoxicity of the NK cells.

### 3.5 Downregulation of MCT4 increased the pH value and decreased the extracellular concentration of lactate

7acc1 selectively and negatively affects MCT4, which leads to a strictly limited efflux of lactate.^31^ To further clarify the link between blocking MCT4 and an increase in the pH value of the acidic microenvironment, we preincubated the 4T1 cells with 7acc1 (0.1, 0.01, 0.001 mmol/L) overnight, collected the supernatant, and then placed the cell supernatant into a 6‐well plate advanced seeded with mouse primary NK cells (1 × 10^6^/well). The measurement of the coculture supernatant pH was performed every 2 hours. Using the Loess regression curve (Figure 4C), we found that the pH values in the 7acc1 (0.1 mmol/L)‐treated group markedly increased over time. The pH values were then tested after transfection of ShMCT4 into the 4T1 cells to block the expression of MCT4. The analysis clearly demonstrated that the higher the ShMCT4 interference efficiency, the greater the improvement in pH values. In the control group, a significant difference was observed during the first day (24 hour) and third day (72 hour) (*P <* *0.05 vs. the control group, Figure 4D). This suggests that blocking MCT4 in breast cancer 4T1 cells may proportionately create an alkaline acid environment that might be beneficial to NK cells.

In previous studies, MCT4 has been shown to export lactate.^15^, ^32^ To further clarify the detailed role that MCT4 plays in the microenvironment, extracellular lactate, which usually exists as a free form in the different concentrations of 7acc1, was measured. We incubated 4T1 cells overnight according to the typical protocol^31^ and then cultivated these cells with 0.1, 0.01, and 0.001 mmol/L 7acc1 dissolved in DMSO in 12‐well plates. The cell supernatant was acquired every 4 hours and then sent for ELISA. We found that the concentration of extracellular lactate declined significantly with time. In addition, as the drug concentration increased, the extracellular lactate significantly decreased (^#^ *P *<* *0.05 vs. the control, Figure 4E).

Lactate is considered a key driver in acidosis‐induced tumor progression. This stimulated two questions: Is lactate an autonomous stimulating metabolite? Does lactate affect the function of NK cells? To assess these question, the sensitivity of NK to lactate was examined. The percentage of specific lysis, which was calculated from the OD values of released calcein in the supernatant, was notably increased after treatment with lactate (0.625, 1.25, 2.5, 10 mmol/L), and the selected concentrations were close to the range of the last ELISA. These results confirm that the NK cells are susceptible to lactate, and lactate can cripple the effect of NK cells on tumor cells in a concentration‐dependent manner (^#^ *P *<* *0.05 vs. the control, Figure 4F).

### 3.6 Depletion of MCT4 induces autophagy to strengthen the defense of NK cells

**Figure 5:**
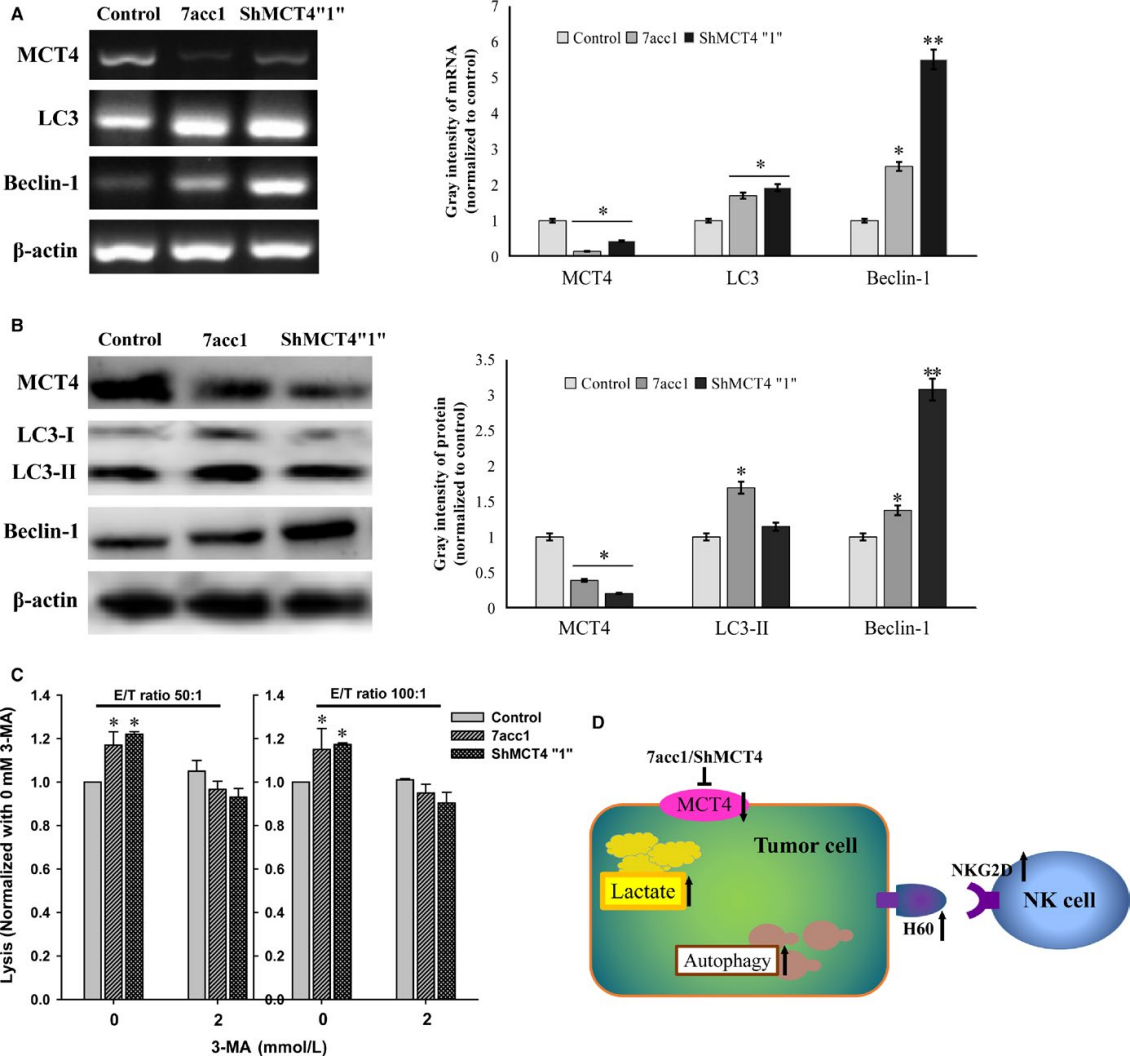
MCT4 deletion induced the compensatory upregulation of autophagy. A, The 4T1 cells treated with 7acc1 (0.1 mmol/L) and ShMCT4”1” 4T1 cell lysates were prepared for immunoblotting for lipidated LC3 and Beclin‐1. B, The 4T1 cells treated with 7acc1 (0.1 mmol/L) and ShMCT4”1” 4T1 cells were exposed to an agent, 3MA (2 mmol/L), that inhibited autophagy. The normalized impact of the agent on lysis was plotted. #P < 0.05. C, The schematic model of this study

It was previously suspected that blockage of MCT4 would trigger autophagy compensation, which would potentially result in greater cytotoxicity in NK cells. It has been confirmed that MCT4 depletion can trigger a metabolic crisis that invokes compensatory mechanisms related to multiple metabolic pathways, including oxidative phosphorylation, macropinocytosis, autophagy, and nutrient‐scavenging mechanisms in pancreatic cancer research.^22^ To determine whether the adaptive biological response of induced autophagy was relative to MCT4 inhibition, we analyzed whether autophagy was increased in the inhibition of MCT4 in 4T1 cells. Because LC3 conversion (LC3‐I to LC3‐II) is generally regarded as an autophagic flux, we tested the expression of endogenous LC3 and the autophagy‐related gene Beclin‐1 by RT‐PCR and Western blot analysis. We observed that the amount of expressed LC3‐II and Beclin‐1 in 7acc1 (0.1 mmol/L)‐treated 4T1 or ShMCT4 4T1 cells were both remarkably increased (Figure 5A). Simultaneously, 3MA (2 mmol/L), an inhibitor of basal autophagy, efficiently crippled the lysis of NK cells at both 50:1 and 100:1 ratios of E/T as expected (^#^ *P *<* *0.05 vs. the control, Figure 5B). Together, these findings indicate that inductive autophagy, as a means of surviving under limited nutrient conditions, rescues the anticancer effect of NK cells.

Based on our study results, we have discovered that the inhibition of MCT4 can induce autophagy to block the lactic exclusion of tumor cells as the extracellular lactate declines and the pH values of the tumor microenvironment rise. In addition, our findings indicate that the NKG2D and H60 were upregulated, which would contribute to the cytotoxicity of NK cells and their ability to kill tumor cells (Figure 5C).

## 4 DISCUSSIONS

The aim of this study was to determine if it is possible to destroy the defiance of cancer cells by targeting glycolytic activity and the associated capacity to export lactate via MCT4, enhancing the possibility of immunological recognition, especially by restoring or enhancing the cytotoxic potency of NK cells, and thereby representing a potential new anticancer therapeutic strategy. Our study revealed that blocking MCT4 can slow tumor growth. This finding is consistent with a previous study, which indicated that intraperitoneal injection of L‐lactate and 3‐hydroxy‐butyrate (a ketone body) increased tumor growth by 2.5‐fold and stimulated the formation of lung metastases by 10‐fold in MDA‐MB‐231 breast cancer in a mouse model.^33^ What we knew about MCT4 was largely based upon experimental studies that investigated how MCT4 impacted the metabolism of a tumor. In a prior study, the metabolic status induced by MCT4 was reported to be an important determinant of the immunosuppressive environment in pancreatic ductal adenocarcinoma.^24^ In another study, MCT4 was noted to functionally alter mitochondrial respiration and induce abnormal glutamine metabolism.^34^ Recent evidence suggests that MCT4 knockdown leads to enhanced intracellular accumulation of lactate and decreased glycolysis in LPS‐treated macrophages, which are essential to a vastly downregulated inflammatory response.^35^

Based on the above, by utilizing different doses of 7acc1 and different efficiencies of ShMCT4 sites designed to interface lactate efflux, it was discovered that alkalizing the tumor microenvironment enhanced NK cell cytotoxicity and further suppressed tumor growth. These results suggested that MCT4‐induced NK cell activation and acidosis of the tumor microenvironment are regulated by an MCT4‐dependent lactate flux in the 4T1 cell line. In addition, we discovered by IHC that the activities of the NK cells also decreased in mouse breast cancer tissues, and this finding is consistent with a previous report.^36^ Based on the principles outlined in the Reverse Warburg Effect hypothesis, glycolysis and lactate can be conducive to the growth of tumor cells.^37^, ^38^, ^39^ It has been previously established that the antitumor activities of NK cells are impaired by hypoxia, acidosis, and glucose metabolic abnormalities.^40^, ^41^ Therefore, repairing the activity of NK cells based on metabolic interventions is considered a reasonable means by which to identify an effective and feasible antitumor intervention. The activity of NK cells has been previously documented to decrease sharply with an elevated concentration of lactate. This finding was confirmed in our study.^42^ Surprisingly, we further discovered that MCT4 depletion, in response to an increase in lactate, activated the compensatory reliance on multiple downstream effector pathways, including autophagy.^22^

For further illuminating the affect to NK cells, we found a marked decline in the number of activated NK cells (NKG2D +cells) infiltrating the tumor using the IHC assay in human breast cancer tissues, and our findings revealed that the metabolic molecule MCT4 was intimately related to the function of NK cells. NKG2D is recognized by NKG2DLs (MICA/B and ULBP1‐6) in humans. Similar results were found for the Rae1, H60, and Mult1 receptors in mice.^43^, ^44^, ^45^ We discovered that blocking MCT4 contributed to the expression of the NKG2D and H60 via the downregulation of pH and eventually improved the ability of NK cells to inhibit the growth of tumors. Thus, the regulation of MCT4 serves as an immune‐editing method to rebuild the immune function of NK cells. Consequently, further research involving other immunity cells and the expression variation of other NKG2DLs in different concentrations of lactate warrants further investigation.

Notably, breast cancer is divided into three subtypes according to the molecular classification: luminal A and B, HER2‐positive and triple‐negative breast cancer (TNBC). The sample of patients was used for the immunohistochemical analysis of TNBC, while those used in the in vivo and in vitro experiments were from the 4T1 cell line, which shows the pathological character of invasive ductal carcinomas in mice. Additionally, 4T1 cells have been used in many mouse breast cancer models for simulating malignant cancer, such as TNBC; however, whether the effect of MCT4 on NK cells occurs in all subtypes of breast cancer still needs more study and we plan to investigate this in the future.

Collectively, these findings revealed that the major protumoral action of the acidified tumor microenvironment is to provide raw materials for the generation of energy in glycolytic tumors via MCT4 activity. These findings also demonstrated that blocking lactic acid export provides an efficient anticancer strategy. Based on the analysis above, we have identified a potential mechanism by which tumor cells can escape immune surveillance, which has provided a potentially new tactic for treating cancer.

## CONFLICT OF INTEREST

None declared.
