# Supplementary material for: Downregulation of MCT4 for lactate exchange promotes the cytotoxicity of NK cells in breast carcinoma
Source: Cancer Med. 2018 Jul 26;7(9):4690–700. doi: 10.1002/cam4.1713 (PMC6143925; doi:10.1002/cam4.1713)
Supplement: Supplementary file 1 [file CAM4-7-4690-s001.pdf]

SUPPLEMENTARY INFORMATION

Down-regulation of MCT4 for lactate exchange promotes the cytotoxicity of NK cells in breast carcinoma

Contents:

Page 2: Supplementary Figures 1(S1-2)

Fig.1 A work model of MCT4

MCT4 is an important protein on the cell membrane, which is structurally able to efflux lactic and pyruvic acids out of the cell, with the function of blocking itself in order to affect the acidity of the tumor microenvironments. It has recently identified that 7-aminocarboxycoumarin (7acc1) could act as a MCT4 inhibitor to block the lactate flux especially in MDA-MB231 cell line [1][2].

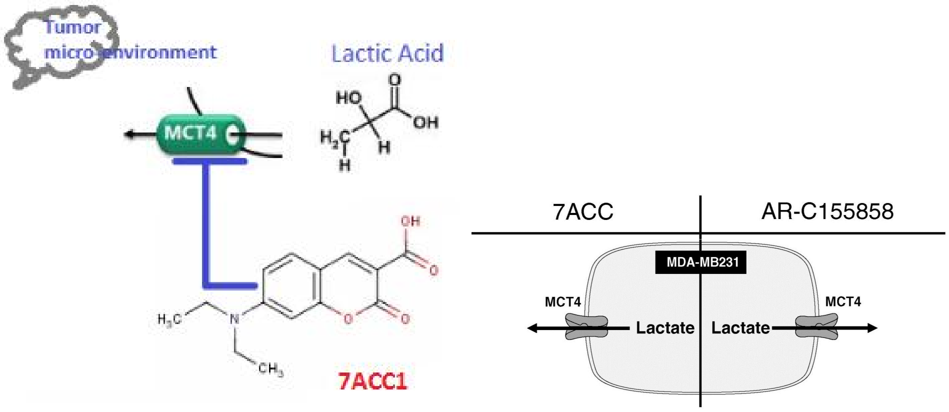

Fig.2 Concentration basis for 7acc1 treatments

It was reported *in vitro* experiment that SiHa, HL60, MDA-MB231 cells were treated by 7acc1 (10 μmol/L) for different time to inhibitor the function of MCT4, and also *in vivo* experiment, 7acc1 was daily injected intraperitoneally on breast cancer mice and tumor growth and mass was tracked [2]. A dose of 3 mg/kg/d was used on the basis of pilot pharmacokinetics studies that had shown that this dosage led to a Cmax > 1 mg/mL and a plasma half-life of 4.5 hours [1]. The MW of 7acc1 is 261.27, which is considered suitable to select a conversion coefficient 300.

In our study, the concentrations of 7acc1 in our study were based on the above data. *In vivo* experiment, we injected 7acc1 intraperitoneal with the concentration gradient: 0.3, 0.03 and 0.003 mg/kg. *In vitro* experiment, we use this following concentration gradient: 0.1, 0.01, and 0.001 mmol/L.

1. Draoui, N., Schicke, O., Fernandes, A., et al. (2013) Synthesis and pharmacological evaluation of carboxycoumarins as a new antitumor treatment targeting lactate transport in cancer cells, *Bioorganic & medicinal chemistry.* **21**, 7107-17.

2. Draoui, N., Schicke, O., Seront, E., Bouzin, C., Sonveaux, P., Riant, O. & Feron, O. (2014) Antitumor activity of 7-aminocarboxycoumarin derivatives, a new class of potent inhibitors of lactate influx but not efflux, *Molecular cancer therapeutics.* **13**, 1410-8.
